# The effect of peripheral dopamine on fracture healing: an experimental study in a rat model

**DOI:** 10.1186/s13018-025-06136-w

**Published:** 2025-07-25

**Authors:** Mücahid Osman Yücel, Raşit Emin Dalaslan, Sönmez Sağlam, Mehmet Arıcan, Zekeriya Okan Karaduman, Banu Turhan, Fatih Demir

**Affiliations:** 1 Faculty of Medicine, Department of Orthopaedics and Traumatology, Duzce University, Konuralp/Duzce, 81620 Turkey; 2 Department of Pediatric Endocrinology, Health Ministry of Türkiye Republic, Ankara Sanatoryum Education and Research Hospital, Ankara, Türkiye; 3 Faculty of Medicine, Department of Pathology, Düzce University, Düzce, Türkiye

**Keywords:** Fracture healing, Dopamine, Levodopa, Bone regeneration, Animal models

## Abstract

**Background:**

Dopamine is a versatile biomolecule that functions as a neurotransmitter, hormone, and immune modulator in the body. Although some anabolic effects of dopamine on bone tissue have been described in the literature, its influence on the complex processes involved in fracture healing remains unclear. This study aimed to evaluate the effects of dopamine on bone healing at the peripheral level.

**Methods:**

Thirty-six male Wistar albino rats were randomly assigned to two groups: a control group with no treatment and a dopamine group that received 12 mg/kg levodopa twice daily via oral gavage following surgery. A standardized femoral fracture was induced under anesthesia in all the rats, which were then fixed with an intramedullary Kirschner wire. Each group consisted of 18 rats, and six rats from each group were randomly sacrificed on postoperative days 15, 30, and 45. The harvested femurs were first evaluated radiologically, followed by biomechanical analysis via a three-point bending test, and finally subjected to histopathological examination.

**Results:**

No significant differences were observed between the groups on days 15 and 30. However, on day 45, histopathological scores were significantly lower in the dopamine group (*p* = 0.015), and biomechanical strength was also lower (*p* = 0.004). Radiological scores were not significantly different between the groups at any time point.

**Conclusion:**

Despite the known anabolic effects of dopamine on bone cells, it may adversely affect fracture healing. The negative impact of dopamine on bone union could be attributed to the multifactorial and complex nature of fracture healing, the dynamics of inflammatory processes, and the cumulative effects of various dopamine receptor subtypes.

## Introduction

Fractures are widely recognized as a common medical issue with significant clinical and economic implications due to the risk of delayed healing or nonunion. Although advances in surgical methods and medical treatments have improved outcomes, complications such as impaired bone healing remain a challenge, and current pharmacological options often show variable effectiveness and may cause side effects [1]. These challenges have led to increasing interest in exploring new biological pathways, including the potential role of dopamine, a molecule known for its diverse functions in the body, in bone health and regeneration.

Like dopamine itself, levodopa, the prodrug of dopamine, acts as a nonselective agonist of all dopamine receptor subtypes (D1–D5) [2]. When administered without enzyme inhibitors, levodopa is rapidly metabolized in the intestines by the enzyme aromatic-L-amino-acid decarboxylase (AADC; EC 4.1.1.28), resulting in peripheral conversion to dopamine [2]. While this increases circulating dopamine levels, the dopamine thus produced cannot cross the blood–brain barrier (BBB), and only approximately 1–3% of levodopa reaches the brain [3]. Therefore, in cases where central dopaminergic effects are desired, levodopa is coadministered with enzyme inhibitors such as carbidopa or benserazide to prevent peripheral metabolism and enhance central availability. This strategy, which is commonly used in Parkinson’s disease treatment, can increase brain levels of levodopa by up to 80% [4]. However, in the present study, levodopa was administered without enzyme inhibitors to investigate the peripheral effects of dopamine.

Dopamine is a fundamental biomolecule with critical roles as a hormone, neurotransmitter, immune modulator, and component of the cholinergic system. Peripheral dopamine release is regulated by various physiological factors, including the intake of precursor amino acids (e.g., tyrosine and phenylalanine), caffeine consumption, psychological stress, physical activity, sleep patterns, sexual behavior, the use of addictive substances, the activation of reward mechanisms, pharmacological agents affecting dopamine metabolism (e.g., L-DOPA, MAO inhibitors), neuroendocrine regulators (e.g., the suppression of prolactin secretion), and the modulatory influence of the gut microbiota on dopamine production [5]. Clinically, dopamine is widely used in the management of acute circulatory failure, such as hemorrhagic shock, as well as in the treatment of several neurodegenerative diseases, most notably Parkinson’s disease.

Owing to its inability to cross the BBB, peripheral and central dopaminergic activities are largely independent of each other [6]. Dopamine exerts contrasting effects on tissue repair depending on the receptor subtype: D1 receptor activation has been shown to promote angiogenesis and wound healing, whereas D2 receptor activation inhibits these processes [7, 8]. These findings indicate that a single hormone may exert opposing effects on physiological processes such as tissue regeneration via different receptor subtypes. Dopamine is known to stimulate osteoblast proliferation and mineralization [9]. Moreover, it suppresses osteoclastogenesis by inhibiting the RANKL signaling pathway via c-Fos and NFATc1 through D2 receptor activation, thereby reducing bone resorption and contributing to skeletal homeostasis [10, 11]. While the role of dopamine in primary bone formation is relatively well established, fracture healing is a distinct physiological process. It proceeds via secondary bone healing, which is characterized by sequential stages: inflammation, soft callus formation, hard callus formation, and remodeling. This complex process is influenced by both systemic and local factors. These local effects have also been investigated in terms of physical interventions. For example, external mechanical stimulation through devices such as intermittent pneumatic compression (IPC) and locally delivered low-intensity pulsed ultrasound (LIPUS) has been reported to enhance bone healing [12, 13]. Moreover, pharmacological agents such as Jintiange have shown potential to enhance bone healing in osteoporotic fractures [14], while systemic factors like stress-induced hyperglycemia have been identified as significant predictors of delayed fracture healing [15]. These findings highlight the need for a comprehensive investigation of both local and systemic factors affecting fracture healing across a broad spectrum.

Given these considerations, we hypothesized that the effects of dopamine on fracture healing warrant a multifactorial approach for evaluation. In this study, we investigated the impact of peripherally active dopamine achieved through the administration of levodopa without enzyme inhibitors on bone healing, with the aim of systematically evaluating its role in the bone regeneration process.

## Materials and methods

### Animal model and experimental design

This experimental study was conducted at the Local Experimental Animal Research and Application Center in accordance with the ARRIVE 2.0 guidelines. Ethical approval was obtained from the Local Animal Experiments Ethics Committee (Decision No: 2024-06-01, dated 17.07.2024).

A total of 36 male Wistar albino rats aged 2 months (skeletally mature) and weighing 200 ± 30 g were acclimatized for one week prior to the experiment under controlled conditions (23 ± 2 °C, 60 ± 5% relative humidity, 12-hour light/dark cycle), with ad libitum access to food and water. The animals were randomly assigned into two groups of 18 rats each. In each group, six rats were sacrificed at postoperative days 15, 30, and 45. The control group received no treatment following surgery. The dopamine group received 12 mg/kg levodopa twice daily via oral gavage from the day of surgery until the day of sacrifice at postoperative days 15, 30, or 45.

In this study, the dopamine treated group was compared only with a negative control group, as no standard agent with a known enhancing effect on fracture healing was administered. Therefore, a positive control group was not included in the experimental design. This decision was made to isolate the specific effects of dopamine and to minimize the significant additional costs of using agents like BMP-2 or bisphosphonates. Similarly, a sham-operated group without fracture was not included, consistent with previous experimental studies investigating dopaminergic mechanisms in wound and bone healing, which also did not incorporate sham or positive control groups [7, 8, 16–18].

### Surgical and postoperative procedures

**Fig. 1:**
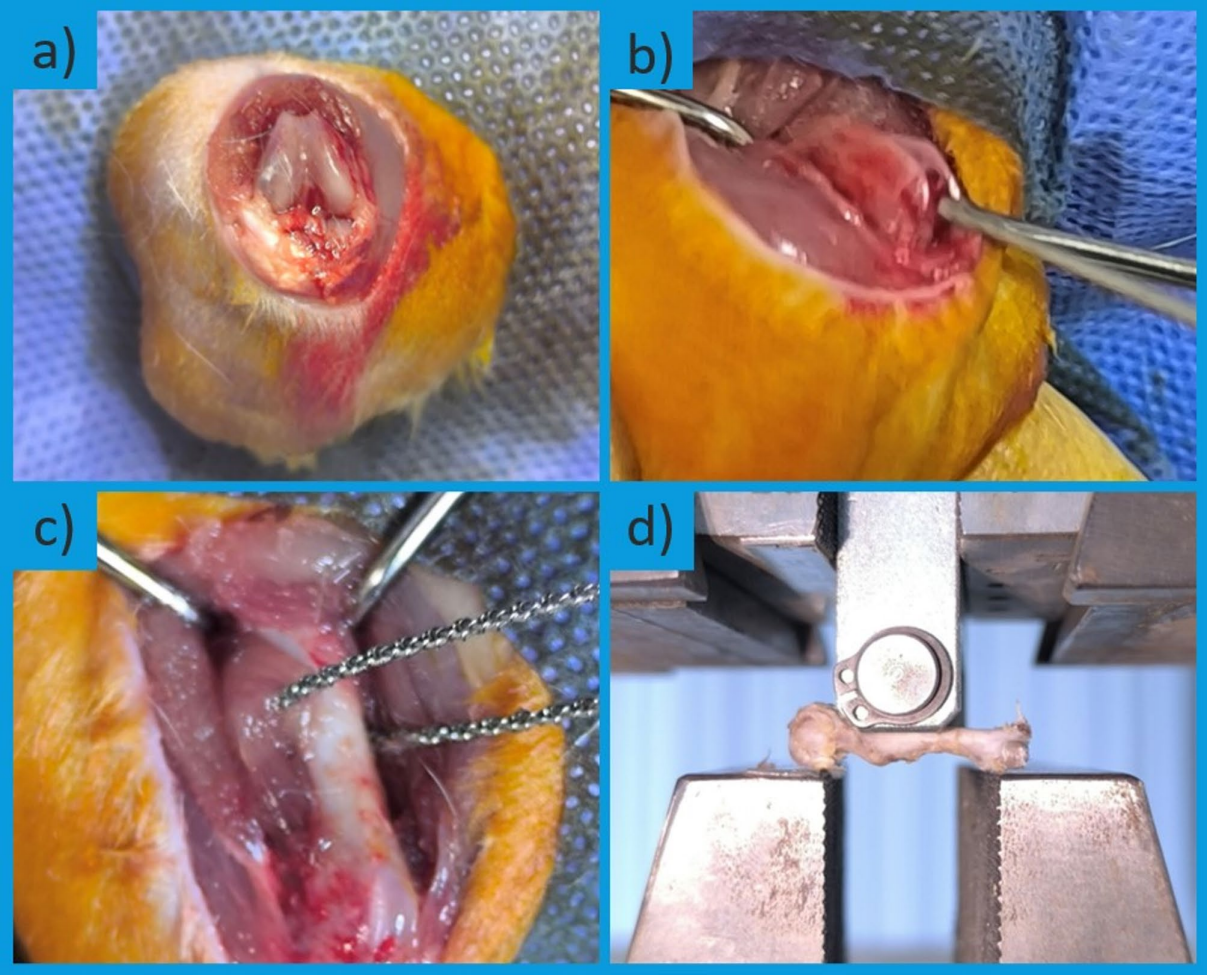
Stages of surgical and testing procedures applied to the rat femur. **a**) Skin incision, **b**) Intramedullary placement of the Kirschner wire, **c**) Osteotomy with a Gigli saw, **d**) Application of three-point bending test

All the rats were anesthetized via the intraperitoneal injection of 90 mg/kg ketamine and 10 mg/kg xylazine. The right thigh was shaved and sterilized. A 1-cm incision was made at the intercondylar region of the femur, and a 1.2 mm Kirschner wire was retrogradely inserted into the medullary canal. A transverse/short oblique fracture was created via a Gigli saw through a secondary incision over the femoral shaft (Fig. 1a–c). Fracture formation was confirmed radiographically. The incision was closed appropriately, and postoperative analgesia was provided via subcutaneous administration of 0.02 mg/kg fentanyl citrate for three days. No postoperative antibiotics were administered to avoid potential confounding effects on bone healing processes, which is consistent with previous experimental fracture studies in rats. Retrograde intramedullary K-wire fixation was selected due to its simplicity and ability to provide adequate stabilization in rat femoral fracture models. Although this method carries minor risks of rotational instability or implant migration, it is generally sufficient for maintaining fracture alignment and supporting the healing process.

On postoperative days 15, 30, and 45, six rats from each group were randomly selected and sacrificed under anesthesia via cervical dislocation. These time points were selected based on previous rat fracture healing studies, where day 15 corresponds to soft callus formation and early endochondral ossification, day 30 reflects predominant hard callus formation, and day 45 represents the beginning of the remodeling phase [19]. Following sacrifice, anteroposterior and lateral radiographs of the femurs were taken. Radiological evaluation was performed via the scoring system defined by Bigham et al. [19]. Femurs were then subjected to biomechanical testing via three-point bending tests to determine fracture site strength in Newtons (N) (Fig. 1d). After biomechanical testing, the callus tissues were processed for histopathological evaluation.

### Histopathological evaluation

At the end of each experimental time point, femoral specimens were harvested and fixed in 10% neutral-buffered formalin. After fixation, the samples were decalcified in 10% formic acid at room temperature for two weeks. Complete decalcification was verified before routine tissue processing and embedding in paraffin. Sections of 4 μm thickness were obtained and stained with hematoxylin and eosin (H&E) for histological assessment.

**Fig. 2:**
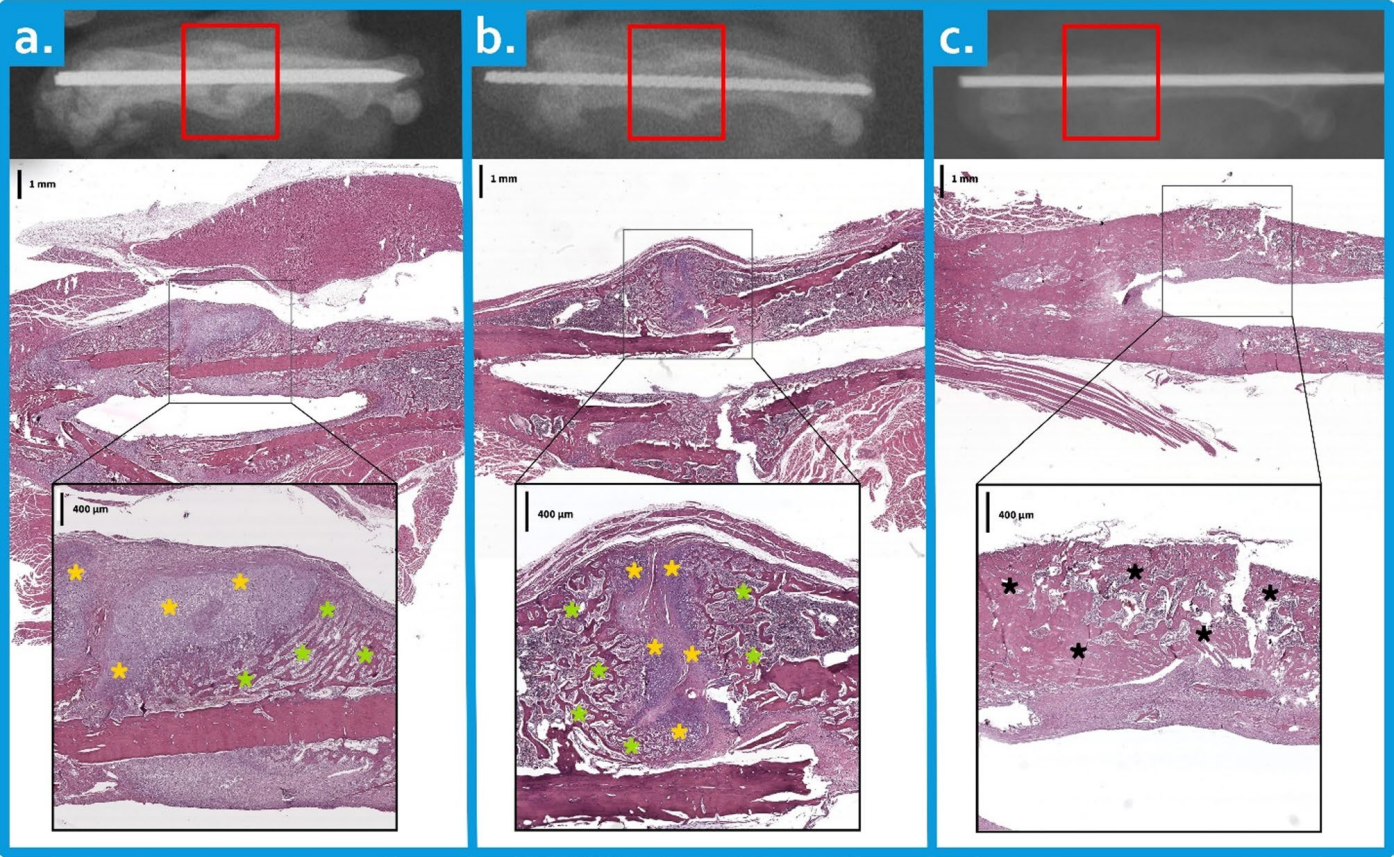
Composite multiscale representation of the histological stages of fracture healing. Each panel (**A**–**C**) shows, from top to bottom: a schematic diagram on the radiograph indicating the region of interest for histological sectioning (red square); a low-magnification histological image (H&E stained) of the decalcified bone section representing this selected region, with an annotated black square indicating the area shown in the bottom image; and a high-magnification histological image providing detailed visualization of the annotated area from the middle image. Scale bars are provided in the upper left corner of each histological image. (a) Mixed cartilage (yellow asterisks) and immature bone (green asterisks), indicating active endochondral ossification consistent with Score 7. (b) Extensive immature bone formation bridging the fracture site (green asterisks) with minimal residual cartilage (yellow asterisks) consistent with Score 8. (c) Dense mature lamellar bone (black asterisks) completely bridging the fracture site, indicating advanced healing consistent with Score 10

Fracture healing was evaluated via the histological scoring system developed by Huo et al. [20]. This numerical scale (1–10) assesses the predominant tissue type at the fracture site: fibrous tissue, cartilage, immature bone, or mature bone (Fig. 2). All samples were evaluated via light microscopy by two independent, blinded pathologists. The average of their scores was used for statistical analysis.

### Radiological evaluation

**Fig. 3:**
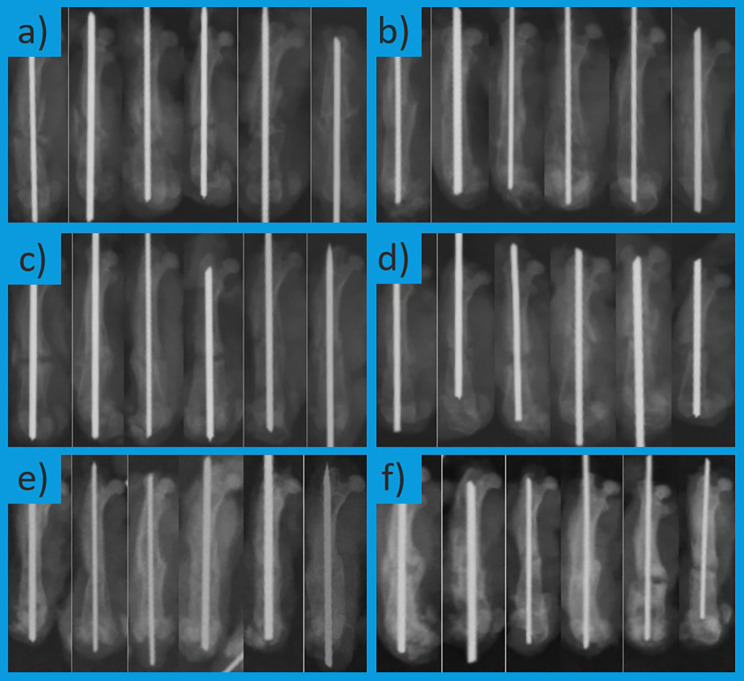
Radiographs of femurs from the control and dopamine groups at different time points. **a**) Day 15 control group; **b**) Day 15 dopamine group; **c**) Day 30 control group; **d**) Day 30 dopamine group; **e**) Day 45 control group; **f**) Day 45 dopamine group

Radiographs of the femurs were obtained on days 15, 30, and 45 from the animals in both groups (Fig. 3). Standard radiographs of the femurs were taken using a digital X-ray system (SEDECAL PLUS LP+, SEDECAL, Madrid, Spain) at 40 kVp and 2 mAs, with a source-to-image distance of 100 cm. Radiographs were evaluated for fracture healing according to the scoring system described by Bigham et al. [19], assessing callus formation, fracture line visibility, cortical continuity, and remodeling. Images were independently assessed by two orthopedic specialists who were not involved in the study. Interobserver agreement was analyzed via the intraclass correlation coefficient (ICC), which yielded a high level of agreement (ICC = 0.88).

### Biomechanical evaluation

Biomechanical analyses were performed on a total of 24 femurs from both groups on days 30 and 45. On the day of sacrifice, each femur was tested immediately without delay. Prior to testing, the intramedullary fixation materials were carefully removed.

Three-point bending tests were conducted via a material testing machine (BESMAK BMT-E Series, Besmak, Ankara, Türkiye) at the institution’s Scientific Technology and Research Center. The distance between the two support points was fixed at 18 mm on the basis of bone length. A compressive force was applied directly over the fracture site at a rate of 3 mm/min until bone failure occurred. The peak force (N) measured immediately before fracture was recorded for each sample. These values were used to compare the mechanical strength between the groups [21].

### Statistical analysis

Descriptive statistics are reported as the means, standard deviations, medians, minimums, and maximums. Comparisons between the dopamine and control groups at each time point (days 15, 30, and 45) were performed via the Bonferroni-adjusted Mann–Whitney U test (α: 0.05/3 = 0.016). All the statistical analyses were conducted via IBM SPSS version 20 (Chicago, IL, USA), and a p value of < 0.05 was considered to indicate statistical significance. Since a normal distribution was not achieved in some subgroups and the sample sizes were small, all group comparisons were carried out via the nonparametric Mann–Whitney U test.

## Results

A total of 36 rats were included in the study, with 18 rats assigned to the dopamine group and 18 to the control group. In both groups, six animals were sacrificed at each of the three time points: days 15, 30, and 45. No implant migration, rotational instability, soft tissue irritation, infection, or mortality was observed in any of the animals during the study.

As shown in Table 1, there was no statistically significant difference in histopathological scores between the dopamine and control groups on days 15 and 30 (*p* > 0.05). However, on day 45, the histopathological scores in the dopamine group were significantly lower than those in the control group (*p* = 0.015). Representative histological images corresponding to some of the histological scores presented in Table 1 are shown in Fig. 2.

**Table 1:** Comparison of histopathological scores between groups

Groups	Mean ± SD	Median (Min-max)	Corrected *p* value
Dopamine(15days)	7.17 ± 0.75	7 (6–8)	*p* = 0.310 ^b^ *p* > 0.05
Control(15days)	6.67 ± 0.81	6.5 (6–8)	
Dopamine(30days)	7.33 ± 0.81	7.5 (6–8)	*p* = 0.041 ^b^ *p* > 0.05
Control(30days)	8.67 ± 1.03	9 (7–10)	
Dopamine(45days)	7.50 ± 1.51	7.5 (6–10)	*p* = 0.015 ^**b**^ *p* < 0.05 ^**b**^
Control(45days)	9.83 ± 0.40	10 (9–10)	

b: Mann Whitney U test (Bonferroni Correction)

As shown in Table 2, there were no statistically significant differences in the radiological scores between the groups at any of the three time points (*p* > 0.05).

**Table 2:** Comparison of radiological scores between groups

Groups	Mean ± SD	Median (Min-max)	Corrected *p* value
Dopamine(15days)	2.83 ± 2.32	2.5 (0–6)	*p* = 0.420 ^b^ *p* > 0.05
Control(15days)	4.00 ± 2.61	4.5 (0–7)	
Dopamine(30days)	6.67 ± 2.42	6.5 (4–11)	*p* = 0.517 ^b^ *p* > 0.05
Control(30days)	7.33 ± 2.16	7.5 (4–10)	
Dopamine(45days)	8.00 ± 1.41	8.0 (6–10)	*p* = 0.035 ^b^ *p* > 0.05
Control(45days)	10.17 ± 1.47	10.5 (8–12)	

b: Mann Whitney U test (Bonferroni Correction)

In terms of biomechanical strength (Table 3), no significant difference was observed between the groups on day 30 (*p* = 0.180). However, on day 45, the force values measured in the dopamine group were significantly lower than those measured in the control group (*p* = 0.004).

**Table 3:** Comparison of Biomechanical strength between groups

Groups	Mean ± SD	Median (Min-max)	Corrected *p* value
Dopamine(30days)	27.70 ± 19.94	18.4 (12.2–59.2)	*p* = 0.180 ^b^ *p* > 0.05
Control(30days)	45.96 ± 13.26	42 (31.2–69.2)	
Dopamine(45days)	43.73 ± 21.06	38.5 (21.2–74.8)	*p* = 0.004 ^**b**^ *p* < 0.01 ^**b**^
Control(45days)	108.86 ± 45.03	96.0 (70.4–191)	

b: Mann Whitney U test (Bonferroni Correction)

**Fig. 4:**
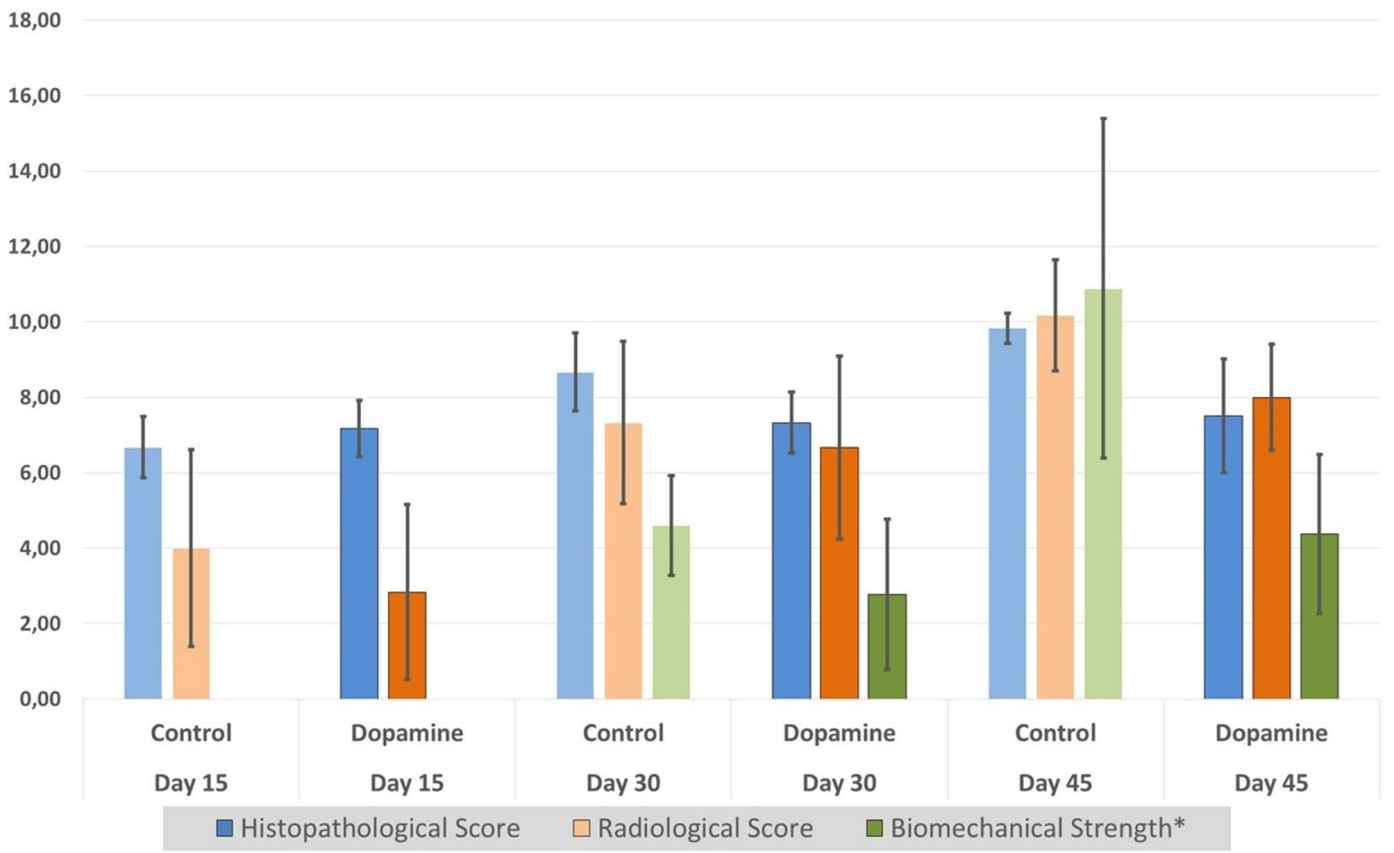
Mean histopathological, radiological, and scaled biomechanical scores at 15, 30, and 45 days in control and dopamine groups. Error bars represent standard deviations. Biomechanical strength values were scaled such that 10 N equals 1 unit to allow graphical comparison and are indicated with an asterisk (*)

In addition to tabular data, the mean histopathological, radiological, and scaled biomechanical scores for both groups at all time points are presented graphically in Fig. 4 to provide a clearer visual comparison of the results.

**Fig. 5:**
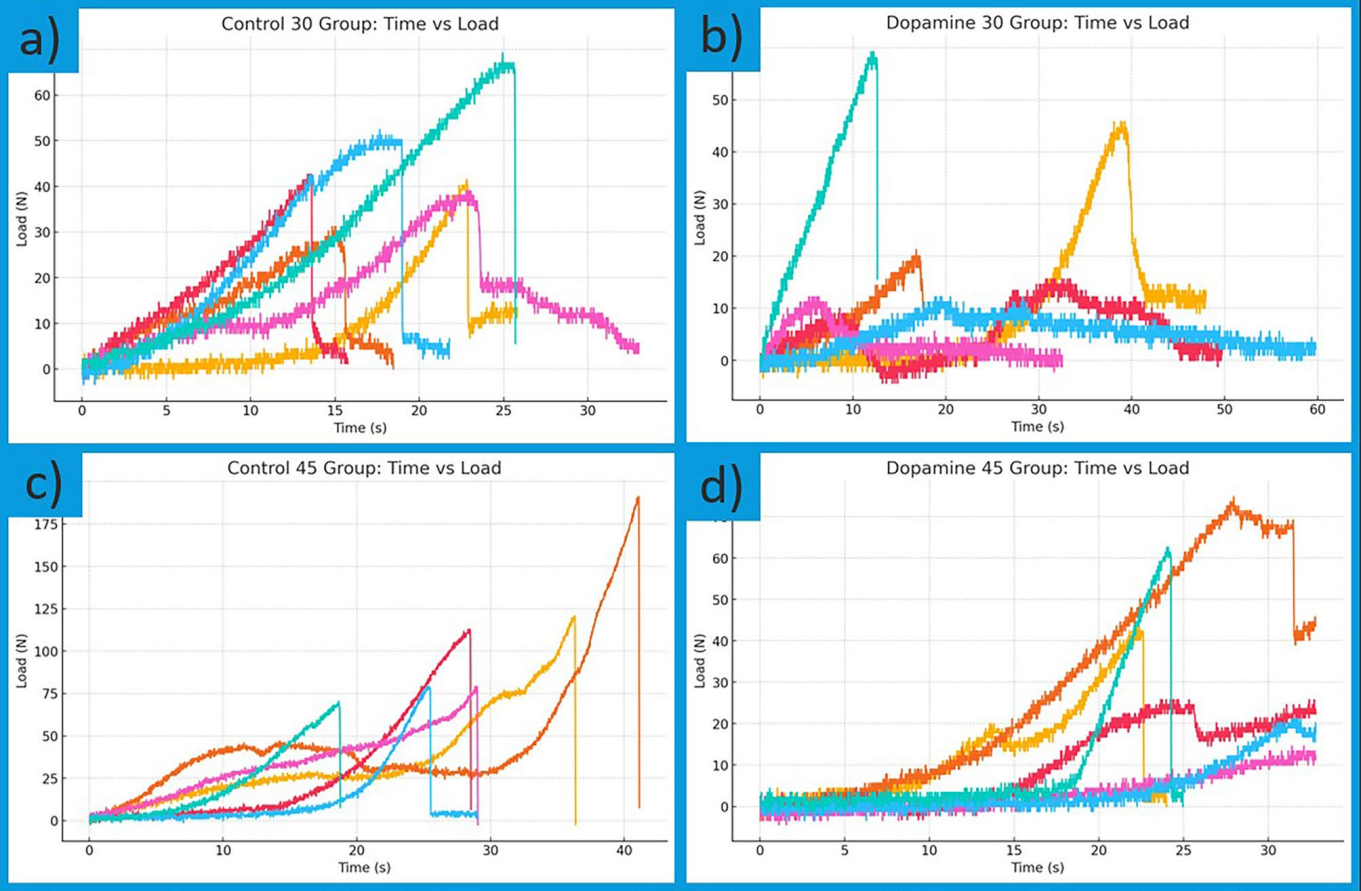
Load‒time curves from three-point bending of femurs in both groups on days 30 and 45. (**a**) Control, day 30; (**b**) Dopamine, day 30; (**c**) Control, day 45; (**d**) Dopamine, day 45

Load‒time profiles from the three-point bending test for femoral samples from both groups on days 30 and 45 are presented in Fig. 5. In the graphs corresponding to the control group (left side), higher fracture load values are observed than those of the dopamine group. Notably, the femurs in the control group on day 45 presented a greater maximum load-bearing capacity. This finding suggests that bone healing in the control group may have been biomechanically stronger.

Overall, while no statistically significant differences were observed between the groups on days 15 and 30 in terms of histopathological or biomechanical parameters, both histological scores and biomechanical strength were significantly lower in the dopamine group compared to the control group on day 45. Radiological evaluations did not reveal significant differences at any time point.

## Discussion

In this study, the effects of systemic dopamine administered via its precursor, levodopa, on an experimental femoral fracture model were investigated. The findings demonstrated that dopamine administration adversely affected both histopathological and biomechanical healing, particularly in the late phase (day 45) (*p* = 0.015, *p* = 0.004).

Dopamine is one of the primary neurotransmitters in the central nervous system and can indirectly influence bone metabolism via the sympathetic nervous system. According to the literature, central dopamine has been shown to enhance osteoclast activity while suppressing osteoblast function, resulting in a reduction in bone mass [22]. On the basis of this mechanism, dopamine at the systemic level may negatively impact bone healing. Indeed, accelerated bone healing has been observed in patients with traumatic brain injury, which is thought to be influenced by neurotransmitters such as dopamine and serotonin, which are centrally released from damaged brain tissue into the peripheral circulation [23]. In our study, to observe the peripheral effects of dopamine on fracture healing, we administered levodopa without enzyme inhibitors.

Several in vitro and animal studies evaluating the effects of dopamine on bone have indicated that dopamine may promote osteoblast proliferation and mineralization [18, 24]. However, these studies focused primarily on primary ossification processes. In contrast, our study assessed the effects of dopamine on secondary bone healing following the creation of a traumatic femoral fracture. Our results revealed that dopamine negatively affected both histopathological and biomechanical healing, particularly on day 45. Although many studies in the current literature emphasize the positive effects of dopamine on osteoblast activity, these findings are based on controlled primary bone formation processes. Secondary bone healing after fracture, however, is a far more complex process involving phases such as inflammation, vascularization, callus formation, and remodeling [1]. Notably, the ability of dopamine to suppress proinflammatory cytokines such as TNF-α and IL-1β may impair the initial inflammatory response, which is crucial for early healing [25]. Additionally, the role of dopamine in skewing macrophages toward the M2 phenotype may hinder the development of transient M1-type inflammation at the fracture site [26]. Moreover, D2 receptor-mediated inhibition of VEGF production may suppress angiogenesis, thus limiting vascularization and impeding oxygen delivery and cell migration ultimately delaying fracture union [7]. Therefore, our study focused not on the effects of dopamine on bone metabolism per se but on its influence on fracture healing.

The biological effects of dopamine vary depending on the dopamine receptor subtypes (D1–D5) involved [27]. These receptors differ in their affinities and activate distinct intracellular signaling pathways. Additionally, they can form homodimers or heterodimers and interact with other receptors, forming mosaic structures and supramolecular organizations [28–30]. As a result of this complexity, dopamine may exert diverse effects even within the same tissue. Indeed, the same dopamine receptor may have proinflammatory effects in one tissue and anti-inflammatory effects in another [31]. D1 receptors support wound healing, whereas D2 receptors may have inhibitory effects [7, 8]. Furthermore, the simultaneous activation of dopamine receptors may lead to synergistic or opposing biological outcomes [17, 32]. Some studies have shown that only D1 receptors play a role in osteogenic differentiation, while other receptor subtypes are not involved [33]. Therefore, given the systemic hormone-like nature of dopamine, evaluating its effects solely on the basis of receptor-level interactions may be insufficient. In this study, the effects of dopamine on bone healing were assessed from a multifactorial perspective rather than exclusively through receptor-based mechanisms.

Indeed, studies in the literature have shown that the effects of certain agents on primary bone formation may not align with their effects on fracture healing. For example, although glucocorticoids, valproic acid, and thyroxine have been shown to exert positive effects on osteoblast differentiation and activity in vitro, they may have detrimental effects on fracture healing in vivo [34–36]. Likewise, bisphosphonates, despite their frequent use in osteoporosis treatment, have been reported to impair fracture healing [37]. Moreover, according to a recent systematic review, pharmacological agents previously considered potentially beneficial for bone healing, such as calcium, vitamin D, and bisphosphonates, have shown no significant positive effects on fracture healing outcomes [38].Similarly, in our study, dopamine, despite its favorable effects on osteoblasts, was found to have negative impacts on bone healing.

In our study, some Kirschner wires appeared relatively long on radiographs, which may have resulted either from distal migration during femoral extraction or intentional placement to achieve sufficient fixation length. However, no soft tissue problems, impairment of joint movements, or negative effects on fracture stability were observed in any of the animals. Therefore, we believe that this situation did not adversely affect fracture healing.

This study has several limitations. Although levodopa was administered without DOPA decarboxylase inhibitors to investigate peripheral effects, it is possible that some of the administered drug crossed the blood–brain barrier and influenced central dopaminergic pathways, which may confound the interpretation of purely peripheral effects. Neither local tissue concentrations nor serum levels of dopamine were measured, which limits the ability to confirm dopamine’s distribution and local effects at the fracture site. Selective agonists or antagonists targeting specific dopamine receptors were not used, making it impossible to determine which receptor subtype played a dominant role. In addition, the study was conducted exclusively in male rats, thereby excluding the potential influence of sex hormones on bone healing. Moreover, differences in the dopamine dosage or route of administration between our in vivo study and previous in vitro experiments should also be considered as a possible explanation for the discrepancies observed in the effects on bone healing. Similarly, a sham-operated group without fracture was not included. This limits the interpretation of dopamine’s baseline skeletal effects independent of fracture healing. Finally, the long-term healing process was not evaluated, as the study focused only on days 15, 30, and 45.

## Conclusions

This study is among the first experimental investigations to evaluate the effects of systemic dopamine levels on secondary bone healing. In this experimental rat femoral fracture model, systemic dopamine administration appeared to have a negative impact on bone healing, particularly in the later stages of fracture repair, as evidenced by lower histopathological scores and reduced biomechanical strength observed on day 45. These findings highlight the need for further research to clarify the mechanisms involved and their potential clinical implications.

## Data Availability

The dataset supporting the findings of this study has been deposited in the Zenodo repository with restricted access: 10.5281/zenodo.15682828. Access to the data will be granted by the corresponding author upon reasonable request.

## Abbreviations

AADC: aromatic-L-amino-acid decarboxylase
BBB: blood–brain barrier
D1-D5: dopamine receptor subtypes
EC: enzyme commission number (EC 4.1.1.28)
H&E: hematoxylin and eosin
ICC: intraclass correlation coefficient
IL-1β: interleukin-1 beta
IPC: intermittent pneumatic compression
LIPUS: locally delivered low-intensity pulsed ultrasound
N: newton
RANKL: receptor activator of nuclear factor kappa-Β ligand
TNF-α: tumor necrosis factor alpha
VEGF: vascular endothelial growth factor
